# Eclampsia*Read before the Brazos Valley Medical Association, at Marlin, Texas, May 14, 1902.

**Published:** 1903-06

**Authors:** W. H. Allen

**Affiliations:** Marlin, Texas


### TEXAS MEDICAL JOURNAL.

ESTABLISHED JULY, 1885.

PUBLISHED MONTHLY.—SUBSCRIPTION $1.00 A YEAR.

Vol. XVIII. AUSTIN, JUNE, 1903. No. 12.

### Original Contributions.

For Texas Medical Journal.

### Eclampsia.*

BY W. H. ALLEN, M. D., MARLIN, TEXAS.

*Eclampsia.*—From a Greek word, meaning "to shine forth." So far as the clinical picture is concerned, there could be no more appropriate name given it. If there is any disease in the category of medicine that shines forth with more force than this, I have yet to find it. And furthermore, any one which demands more skillful and prompt attention.

My idea in medical nomenclature has always been that the name should always represent, in a measure, or in full, some exact and definite pathological lesson, or condition.

That eclampsia is a toxemia we can not dispute, but that this toxemia is always accompanied with, or that albumen or urea is the cause of the trouble, I am loth to accept. With the few cases that have come under my observation, 50 per cent. have had no trace of albumen, nor has the urine contained tube casts. I have never been able to make post-mortem to examine kidneys.

All diseases must originate from one of two sources, namely: From conditions originating from without and introduced in the body. Or from those originating within the body. Much has been written about auto-intoxication, and many are the theories advanced as to the toxins formed; their mode of formation; their elimination, retention, and their effect upon the human organism. Some one has rightly said that the living organisms in the normal, as in the pathological state, is a receptacle and laboratory of poisons. My idea is that in this trouble the poison is absorbed from the placental area of uterus. One of the principal proofs of

*Read before the Brazos Valley Medical Association, at Marlin, Texas, May 14, 1902.

this position is one we have all observed, and that is the complete cessation, or diminution of attacks after delivery, and again on examination of placenta in such trouble, it is always in bad condition, showing degeneration.

The albumen and accompanying nephritic trouble is due to the poison in the blood, and irritating effects of this poison on kidney structures. The pressure theory can be very easily overcome by the fact, as we all have observed, that in uterine or ovarian tumors of very large size we do not have such phenomena.

It is the culminating of certain conditions. The great predisposing cause is the retention within the body, absorbed from placental uterus, the products of metabolism and decomposition.

As with the kidney, just so with the liver. The disturbed liver function is not a cause, but a result of eclamptic condition. In the body of every pregnant woman, but especially toward the end of gestation, there circulates increased quantities of incomplete oxidized substances—leucomaines.

This abnormally large amount of toxic substance, with the addition of more from diseased placental mass being constantly taken up in the circulation, very easily disturbs the physical equilibrium of subject. That eclampsia occurs more frequently in primipara than in multipara, is due to the nervous equilibrium being more highly sensitized and more easily disturbed in primipara.

FROM SOJOUS' ANNUAL.

Careful histological studies made of the various organs in a large number of puerperal cases. In the vessels were found large multinucleated cells, which were considered to be cells derived from the placenta, and also multiple capillary thrombosis From these facts the conclusion drawn that the disease is essentially due to the pressure in the blood of a coagulating ferment, formed either by the degeneration of the few placental cells found in the blood or by the degenerative changes in the placenta itself. Eclampsia is not common in women the subjects of chronic kidney disease before pregnancy.

The toxins causing uremia are varied and numerous. In eclampsia the urine is less toxic than normal, while the blood serum is more toxic. The fetus is an additional source of waste products, and an additional cause of danger to the mother. Any miasmatic disease will have a tendency to aggravate this trouble; patients who have been living in swamps are more violently affected.

TREATMENT.

Can be divided into (1) prophylactic, (2) medical, (3) surgical.

Too much stress can never be put on the fact that all pregnant women should be closely looked after during the entire period of gestation, and more especially during the last three months of this period. And by looking after her does not mean to take any member of the family's statement that she is doing well. An edema of feet or hands, scanty urination, pains about the head, more especially about the back of the head or up and down the spine, spots before the eyes, should always be attended to at once.

Diet should be guarded. We should direct more preferably a milk diet, or something that can be easily assimilated, and contain plenty of liquids. Eliminative organs should be watched and kept in perfect order. I don't care for many remedies. Chloroform inhalations during a paroxysm—hypodermic injections of Norwood's Tr. V.V. Begin twenty minutes, keeping circulation below 60; probably repeating from two to six hours, as necessary. I never use pilocarpine.

I consider blood-letting and saline infusions to be the sheet anchor. I have never yet bled a patient for eclampsia but that she got well. This is perfectly rational and reasonable treatment; diminishing the amount of poison in blood. If I had a severe case, I would not hesitate to bleed in one arm and throw in salt solution in the other; by this means thoroughly wash out the circulation. This will be true if the trouble is due either to ptomaines, bacteria, or whatever may be the poison.

If this course be pursued, I do not consider the suggestion of induction of labor necessary—am sure I would not advise it at all. I do not consider that the expulsion of fetus stops spasms only by removing the area of poison absorption. The loss of blood usually accompanying delivery does more good than the removal of pressure of child.

In conclusion, if I can impress upon my hearers the importance of reviving the old-time treatment of blood-letting in every case of eclampsia, whether mild or severe, I will consider that I am well paid for the reading of this paper. It may seem barbarous, or that I am advocating a treatment that we have, all of us, at some time or another, thought that numbers of lives have been sacrificed by the practice, but I am fully convinced that all cases can be relieved by the practice.

